# Delayed care in carotid-cavernous fistula due to the Covid-19 pandemic

**DOI:** 10.5935/0004-2749.20220057

**Published:** 2025-08-21

**Authors:** Facundo Urbinati, Francisco Zamorano-Martin, Maria Garcia-Lorente, Rahul Rachwani-Anil, Elisabet Martin-Gonzalez, Carlos Rocha-de-Lossada

**Affiliations:** 1 Department of Ophthalmology, Regional University Hospital of Malaga, Malaga, Spain; 2 Department of Ophthalmology, University Hospital of Virgen de las Nieves de Granada, Granada, Spain

**Keywords:** Carotid-cavernous fistula, Covid-19, Retinal vein thrombosis, Fístula carotidocavernosa, Covid-19, Oclusão da veia retiniana

## Abstract

Direct carotid-cavernous fistula is a high-flow communication between the internal carotid artery and the cavernous sinus that requires early transarterial embolization for its resolution. We report a case of a patient with a direct carotid-cavernous fistula *who subsequently developed* a central retinal vein thrombosis due to a delay in treatment related to the health collapse experienced in the first months of the Covid-19 pandemic in Spain.

## Abstract (pt)

A fístula carótido-cavernosa direta é uma comunicação de alto fluxo entre a artéria carótida interna e o seio cavernoso que requer embolização trans-arterial precoce para sua resolução. É relatado aqui o caso de um paciente com fístula carótido-cavernosa direta *que posteriormente desenvolveu* uma trombose da veia central da retina devido a um atraso no tratamento relacionado ao colapso de saúde experimentado nos primeiros meses da pandemia de Covid-19 na Espanha.

## INTRODUCTION

Carotid-cavernous fistula (CCF) results from abnormal communication between arteries and veins within the cavernous sinus^(1)^. CCF can be classified as direct or indirect, which are separate conditions with different etiologies. Direct CCF is a high-flow communication between the internal carotid artery (ICA) and the cavernous sinus, and it requires transarterial embolization for its closure and resolution^(2,3)^. In the current context of the Covid-19 pandemic, the Spanish Health System has been overwhelmed. Because of delays in the diagnosis and treatment of pathologic conditions, there has been an increase in their complications^(4-6)^.

## CASE REPORT

A 71-year-old woman with no relevant medical history was referred to our emergency service on March 10, 2020, complaining of bilateral eyelid inflammation and diplopia for 2 weeks.

A complete standard ophthalmology examination was performed. The patient had visual acuity (VA) on the Snellen scale of 20/40 in the right eye (RE) and 20/20 in the left eye (LE). The pupillary light reflex was normal. *O*cular motility showed *r*estriction of adduction and supraduction in the RE. Slit-lamp *biomicroscopy* of the *anterior segment* showed bilateral soft eyelid edema and bulbar conjunctival hyperemia with chemosis, along with 2 mm of proptosis of the RE. Intraocular pressure (IOP) by Goldman tonometry was 14 mmHg in the RE and 15 mmHg in the LE. The fundus examination was *within normal limits*.

**Figure 1:**
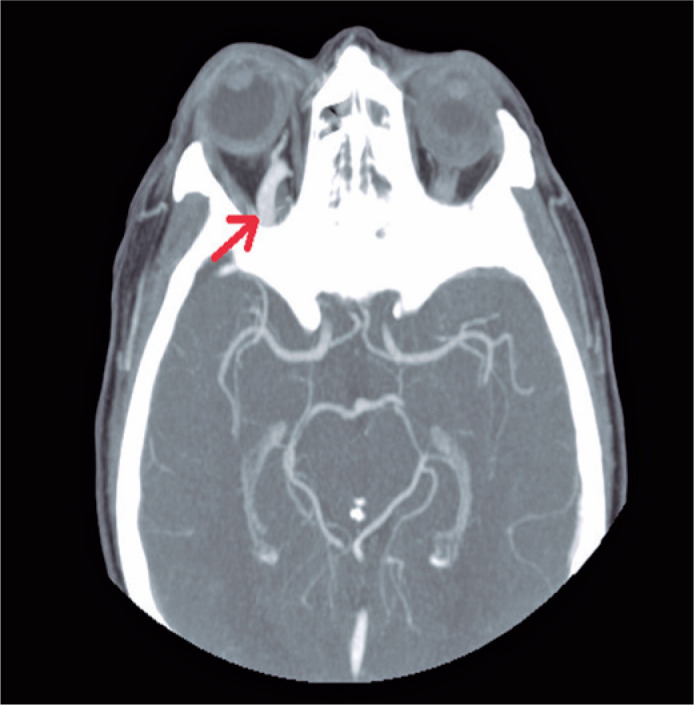
Computerized axial tomography with contrast showing an increase in the caliber of the right superior ophthalmic vein (red arrow) accompanied by ipsilateral exophthalmos.

After these findings, cranial and orbital contrast computed tomography was performed. Computed tomography showed a marked increase in the caliber of the right superior ophthalmic vein, compatible with an arteriovenous fistula (Figure 1). A consultation was made with the Radiology Department, which indicated a preferential evaluation for a possible transarterial embolization. One week later, the appointment was postponed indefinitely because of the declaration of state of alarm due to Covid-9.

One month after the first visit, the patient returned to our emergency department for worsening of VA in the RE. On examination, the patient had VA of 20/200 in the RE and 20/20 in the LE. In addition, she presented a relative afferent pupillary defect in the RE and maintained proptosis in that eye. Ocular motility and anterior segment biomicroscopy findings were similar to those in the previous visit. The IOP was 16 mmHg in the RE and 17 mmHg in the LE. Funduscopic examination revealed flame-shaped hemorrhages in all four quadrants, venous tortuosity, and macular edema, an image suggestive of central retinal vein thrombosis. Against this background, the Radiology Department was contacted again to speed up the intervention process, which was finally performed in the following week.

## DISCUSSION

The Covid-19 pandemic has put some health systems under immense pressure, and in order to prioritize, there has been a delay in the diagnosis of other pathologies that could be considered equally severe and urgent.

CCF is an abnormal communication between arteries and veins through the cavernous sinus. It can be classified as direct or indirect (or dural)^(1)^. Direct fistulas (type A) are characterized by a communication between the ICA and the cavernous sinus, are high-flow, and are generally caused by trauma or ruptured aneurysms^(2)^. Indirect fistulas (type B) are normally low-flow and are communications between the cavernous sinus and meningeal arterial branches of the ICA^(7)^. The symptoms depend on the type of fistula and include proptosis, arterialization of the conjunctival and episcleral vessels, strabismus due to ocular motor nerve paralysis, increased IOP, stasis retinopathy, central retinal vein thrombosis, and optic neuropathy^(8)^.

Endovascular management is the mainstay of treatment of direct CCF, whereas conservative management is often employed first for indirect FCC because up to 70% close spontaneously^(3)^. In our case, delay in management of the fistula resulted in the development of a thrombosis of the central retinal vein, with consequent worsening of the visual prognosis.

During the lockdown period, there has been a decrease in the number of ophthalmological emergencies, highlighting a decrease in the number of patients affected by retinal detachment (RD) and uveitis, pathologies whose prognosis worsens with late management^(6)^. Subsequent studies have shown how the delay in the diagnosis of these pathologies has resulted in an increase in the number of RD cases with associated macular detachment at the time of diagnosis^(5)^.

In other medical specialties, there has been an increase in the number of urgent surgeries and severe *decompensations* of *chronic diseases.* For instance, in the case of colorectal cancer, delay in performing the intervention has led to elective surgeries becoming urgent^(9)^. Similarly, there has been a decrease in the number of hospitalizations for heart failure, and the patients who were admitted had higher levels of N-terminal pro-brain natriuretic peptide (NT-proBNP), indicating a higher degree of congestion and suggesting that they were admitted to the hospital later^(10)^.

CCF is a pathology that requires interdisciplinary management and prompt resolution to avoid complications that worsen the patient’s visual prognosis. Our case highlights the importance of not postponing the treatment of this pathology as well as other disorders not related to coronavirus disease, despite the situation in the health system.
